# Genetic polymorphisms associated with psoriasis and development of psoriatic arthritis in patients with psoriasis

**DOI:** 10.1371/journal.pone.0192010

**Published:** 2018-02-01

**Authors:** Nikolai Dyrberg Loft, Lone Skov, Mads Kirchheiner Rasmussen, Robert Gniadecki, Tomas Norman Dam, Ivan Brandslund, Hans Jürgen Hoffmann, Malene Rohr Andersen, Ram Benny Dessau, Ann Christina Bergmann, Niels Møller Andersen, Mikkel Kramme Abildtoft, Paal Skytt Andersen, Merete Lund Hetland, Bente Glintborg, Steffen Bank, Ulla Vogel, Vibeke Andersen

**Affiliations:** 1 Department of Dermatology and Allergy, Herlev and Gentofte Hospital, University of Copenhagen, Hellerup, Denmark; 2 Department of Dermatology, Aarhus University Hospital, Aarhus, Denmark; 3 Department of Dermatology, Bispebjerg Hospital, Copenhagen, Denmark; 4 Dermatology Clinic, Nykoebing Falster, Denmark; 5 Department of Clinical Immunology and Biochemistry, Lillebaelt Hospital, Vejle, Denmark; 6 Institute of Clinical Medicine, Aarhus University, and Department of Respiratory Diseases and Allergy B, Aarhus University Hospital, Aarhus, Denmark; 7 Department of Clinical Biochemistry, Herlev and Gentofte Hospital, University of Copenhagen, Hellerup, Denmark; 8 Department of Clinical Microbiology, Slagelse Hospital, Slagelse, Denmark; 9 Focused research unit for Molecular Diagnostic and Clinical Research, IRS-Center Soenderjylland, Hospital of Southern Jutland, Aabenraa, Denmark; 10 Zitelab Aps, Copenhagen, Denmark; 11 Department of Microbiology and Infection Control, Serum Institute, Copenhagen, Denmark; 12 Department of Clinical Medicine, Faculty of Health and Medical Sciences, University of Copenhagen, Copenhagen, Denmark; 13 The DANBIO registry and Copenhagen Center for Arthritis Research (COPECARE), Center for Rheumatology and Spine Diseases, Centre of Head and Orthopaedics, Rigshospitalet, Glostrup, Denmark; 14 Department of Rheumatology, Herlev and Gentofte Hospital, Hellerup, Denmark; 15 National Research Centre for the Working Environment, Copenhagen, Denmark; 16 Institute of Molecular Medicine, University of Southern Denmark, Odense, Denmark; 17 OPEN (Odense Patient data Explorative Network), University of Southern Denmark, Odense, Denmark; Universita degli Studi di Roma Tor Vergata, ITALY

## Abstract

**Background:**

Psoriasis (PsO) is a chronic inflammatory disease with predominantly cutaneous manifestations. Approximately one third of patients with PsO develop psoriatic arthritis (PsA), whereas the remaining proportion of patients has isolated cutaneous psoriasis (PsC). These two phenotypes share common immunology, but with different heredity that might in part be explained by genetic variables.

**Methods:**

Using a candidate gene approach, we studied 53 single nucleotide polymorphisms (SNPs) in 37 genes that regulate inflammation. In total, we assessed 480 patients with PsO from DERMBIO, of whom 151 had PsC for 10 years or more (PsC10), 459 patients with PsA from DANBIO, and 795 healthy controls. Using logistic regression analysis, crude and adjusted for age and gender, we assessed associations between genetic variants and PsO, PsC10, and PsA, as well as associations between genetic variants and development of PsA in PsO.

**Results:**

Eleven polymorphisms in 10 genes were nominally associated with PsO and/or PsC and/or PsA (*P* < 0.05). After correction for multiple testing with a false discovery rate of 5%, two SNPs remained significant: *TNF* (rs361525) was associated with PsO, PsC10, and PsA; and *IL12B* (rs6887695) was associated with PsO.

**Conclusion:**

Among a cohort of Danish patients with moderate-to-severe psoriasis, two SNPs in the *IL12B* and *TNF* genes were associated with susceptibility of psoriasis. None of the SNPs were specifically associated with isolated cutaneous psoriasis or psoriatic arthritis.

## Introduction

Psoriasis (PsO) is a chronic inflammatory disease that affects approximately 2–4% of the western world’s population [1]. Approximately one third of patients with PsO develop psoriatic arthritis (PsA) [2], while the remaining patients have isolated cutaneous psoriasis (PsC). PsA and PsC share common immunology, in which the interleukin (IL)-23/IL-17 axis plays a critical pathogenic role [3]. Other important cytokines include tumor necrosis factor alpha (TNF-α), interleukin (IL)-12, IL-22, and interferon gamma (IFN-γ) [4, 5], with NFκB being a crucial mediator in the pathogenesis [6].

Epidemiological studies suggest stronger heritability for PsA than for PsC (9) which indicates that there could be individual risk loci for these two disease entities. Identification of such loci could potentially serve as novel drug targets and possibly improve patient outcomes by facilitating earlier detection of PsA, thereby possibly preventing irreversible joint destruction [7–9]. Indeed genetic differences related to the human leukocyte antigen (HLA) class I region of the major histocompatibility complex (MHC) have been well established. Variants of HLA-B, namely HLA-B*27, have been proven to confer risk of PsA [10], while HLA-C*06 has been demonstrated to hold a specific risk for PsC [10–14].

Only a limited number of studies have evaluated genetic loci specifically associated with PsA, PsC and the differences between PsA and PsC. To date only 15 polymorphisms in twelve genes outside the MHC region have been reported to render differences in susceptibility between the two phenotypes, including *IL23R* (rs2201841, rs12044149) [11, 12, 15], *FBXL19* (rs10782001) [16], *IL12B* (rs2082412) [11, 17], *ZNF816A* (rs9304742) [17], *CCR2* (rs1799864) [18], *CSF2* (rs715285) [19], *PTPN22* (rs2476601) [12, 20], *IL13* (rs1800925, rs20541, rs848) [21–23], *TNFRSF9* (rs4908742) [12], *LCE3A* (rs10888503) [12], *TNFAIP3* (rs9321623) [12], and *KIR2DS2* [24]. Among these, only associations to *PTPN22* (rs2476601) [12, 20], *IL23R* (rs2201841, rs12044149) [11, 12, 15], *IL12B* (rs2082412) [11, 17], and *IL13* (rs1800925, rs20541, rs848) [21–23] have been replicated, while a large scale genome wide association study (GWAS) failed to reproduce the associations for the polymorphisms in *IL13* and *IL12B* [12].

In order to potentially add to the number of loci outside of the HLA region that confer risk specific to PsA and PsC, this study examined functional single nucleotide polymorphisms (SNPs) in genes involved in regulation of the NFκB pathway (*CD14*, *LY96*, *MAP3K14*, *NFKB1*, *NFKBIA*, *SUMO4*, *TIRAP*, *TLR1*, *TLR2*, *TLR4*, *TLR5*, and *TLR9*), cytokines regulated by NFκB (*IL1B*, *IL1RN*, *IL6*, and *IL10*), TNF-α signaling (*TNFA*, *TNFAIP3*, and *TNFRSF1A*), IFN-γ signaling (*IFNG*, *IFNGR1*, *IFNGR2*, *JAK2*, and *TBX21*), the IL-23/IL-17 axis (*IL12B*, *IL12RB1*, *IL17A*, and *IL23R)*, and other genes involved in regulation of inflammation (*CARD8*, *IL4R*, *IL6R*, *IL18*, *NLRP1*, *NLRP3*, *PPARG*, *PTPN22*, and *TGFB1*). These were assessed for association with PsO, PsC, and PsA and compared to the general population, and furthermore they were examined for association with development of PsA in PsO.

## Materials and methods

### Cohort

As described by *Loft et al*. a Danish cohort comprising 480 patients, registered in the national database DERMBIO, diagnosed with PsO was established [25]. Among these patients, 147 had an additional diagnosis of PsA recorded in DERMBIO [25]. Patients registered in DERMBIO have all received biological therapy in a dermatological setting, thus all patients had moderate-to-severe involvement of skin. We identified a subgroup of 151 patients with PsC who had been retrospectively followed for ≥10 years prior to initiation of biological treatment (PsC10) and who did not have an additional PsA diagnosis in DERMBIO. This group represented a group of patients with isolated cutaneous psoriasis who were unlikely to develop PsA, as the majority of patients with PsO, who develop PsA do so within the first 10 years after diagnosis [26].

Additionally, a PsA cohort was established. This included 459 patients diagnosed with PsA according to their treating rheumatologist in DANBIO, an independent Danish nationwide quality registry for patients treated in a rheumatology setting with disease modifying anti rheumatic drugs (DMARDs) including biological (b)DMARDs [27]. There was no overlap of included patients in DERMBIO and DANBIO.

Patients were identified by linking the unique personal identification number of Danish citizens (CPR number) from blood clot samples, sent for *Mycobacterium tuberculosis* (TB) screening, with DANBIO and DERMBIO. Screening for TB before the initiation of biological therapy is a routine element of care in Denmark. Blood clot samples were obtained from: the Department of Biochemistry, Hospital of Lillebaelt (Vejle, Denmark); the Department of Biochemistry, Hospital of Slagelse (Slagelse, Denmark); the Department of Clinical Biochemistry, Herlev and Gentofte Hospital (Hellerup, Denmark); the Department of Respiratory Diseases B and the Department of Clinical Microbiology, Aarhus University Hospital (Aarhus, Denmark); and Statens Serum Institut (Copenhagen, Denmark) from September 2009 through July 2015, as described earlier [25, 28–31]. A previously described group of 795 healthy blood donors recruited from Viborg, Denmark was used as control group [32].

### Ethical considerations

The study was conducted in accordance with the Declaration of Helsinki and was approved by Regional Ethics Committees of Southern (S-20120113) and Central (M-20100153) Denmark and the Danish Data Protection Agency of Southern (RSD: 2008-58-035) and Central (RM: J. 2010-41-4719) Denmark. The Ethics Committees gave exemption from obtaining written informed consent, because information had no health-related impact.

### Genotyping

For the patients, DNA was extracted from cryopreserved blood clots by using the Maxwell 16 Blood purification kit (Promega, Madison, Wisconsin, USA), as described by *Bank et al*. [33]. DNA from the healthy controls was extracted according to the manufacturers’ instructions from EDTA-stabilized peripheral blood using either a either PureGene (Qiagen, Hilden, Germany) or Wizard Genomic (Promega, Madison, Wisconsin, USA) DNA purification kit [32].

A candidate gene approach was chosen, with focus on genes involved in the NFκB pathway, TNF-α signaling, the IFN-γ pathway, and the IL-23/IL-17 axis, as described earlier [28–30]. In addition, other genes involved in regulation of inflammation including the inflammasome were assessed [34]. In short, relevant genes were identified by searching pathway databases (http://www.genome.jp/kegg/pathway.html and http://www.wikipathways.org/index.php/WikiPathways) and SNP candidates were identified by searching PubMed with *“polymorphism AND Gene name AND (reporter gene OR luciferase OR ELISA OR RT-PCR OR flow cytometry OR EMSA)”*. The SNPs were selected based on the reported functionality or associated with autoimmune diseases (S1 Table). Additionally, SNPs or genes associated with response to biological treatment of either inflammatory bowel diseases or rheumatoid arthritis were included [28–30].

The polymorphisms were genotyped with Competitive Allele-Specific Polymerase chain reaction (KASP™) by LGC Genomics (LGC Genomics, Hoddesdon, United Kingdom) (http://www.lgcgenomics.com/). Genotype distributions for healthy controls and phenotypes are presented in S2 Table.

Linking disequilibrium (LD) was calculated using SNP Annotation and Proxy Search (SNAP) software (http://archive.broadinstitute.org/mpg/snap/) using as reference the Central Europeans in the 1,000 Genomes [35].

As a quality control, all SNPs were replicated for 94 randomly selected samples, yielding >99% identical genotypes.

### Power analysis

At the 5% significance level, and a minor allele frequencies (MAF) of 0.05, 0.25, and 0.45 there is > 80% power for detecting a dominant effect with an odds ratio (OR) of 1.7, 1.4, and 1.5, respectively, for PsO, an OR of 2.0, 1.7, and 1.9, respectively, for PsC10, an OR of 1.7, 1.4, and 1.5, respectively, for PsA, and for PsA in patients diagnosed with PsO an OR of 1.8, 1.5, and 1.6, respectively (Table 1). The Genetic Power Calculator [36] was used for power calculations, setting ‘prevalence’ of PsO to 2%, of PsC10 to 1.4%, of PsA to 0.6%, and of PsA in PsO to 30%, D-prime to 1, type 1 error rate to 0.05, and number of cases and control:case ratio was based on data described in Table 2.

**Table 1:** Minimum effect size in which we had > 80% power at 5% significance level and a minor allele frequency (MAF) of 0.05, 0.25, and 0.45 for each phenotype.

Phenotype	‘Prevalence’	MAF = 0.05	MAF = 0.25	MAF = 0.45
PsO	2.0%	1.7	1.4	1.5
PsC10	1.4%	2.0	1.7	1.9
PsA	0.6%	1.7	1.4	1.5
PsA in PsO	30%	1.8	1.5	1.6

**Abbreviations:** MAF, minor allele frequencies; PsO, psoriasis; PsA, psoriatic arthritis; PsC, cutaneous psoriasis; PsC10, patients with PsC followed for 10 years or more. The Genetic Power Calculator [36] was used for power calculations, setting ‘prevalence’ according to corresponding phenotype, D-prime to 1, type 1 error rate to 0.05 and number of cases and control:case ratio was based on data described in Table 2.

**Table 2:** Characteristics and demographics of patients with psoriasis, psoriatic arthritis, cutaneous only psoriasis, and healthy controls.

	PsO (n = 480)	PsC10 (n = 151)	^#^PsA (n = 459)	Controls (n = 795)
**Gender, n (%):**				
Male	285 (59)	99 (66)	209 (46)	411 (52)
Female	195 (41)	52 (34)	250 (54)	384 (48)
**Age, years:**				
Mean (SD)	44 (14)	45 (14)	46 (13)	43 (12)
**Age at diagnosis, years:**				
Mean (SD)	24 (14)	21 (13)	41 (13)*	-
**Disease duration, years:**				
Mean (SD)	20 (13)	25 (12)	5 (7)*	-

**Abbreviations:** PsO, psoriasis; PsC, cutaneous psoriasis; PsC10, patients with PsC followed for ≥10 years; PsA, psoriatic arthritis.

^#^patients registered in DANBIO with PsA.

*Diagnosis of PsA

### Statistical analysis

Assuming a dominant model, we compared genotype distributions for the following groups: patients with PsO and PsC10 from DERMBIO compared with healthy controls using logistic regression analysis; patients with PsA from DANBIO compared with healthy controls; patients with PsA from DANBIO compared to patients with PsC10 from DERMBIO. Odds ratios unadjusted and adjusted for age and gender are reported (S3 and S4 Tables). Correction for multiple testing by controlling the false discovery rate (FDR) at 5% was performed [37]. Additional computation of FDR-adjusted p-values (q-values) based on all *P*-values presented in S3 and S4 Tables were performed. Q-values describe the estimated proportion of false positives among associations equal to, or more extreme than, the observed association.

Chi-square test was used to test deviations from Hardy-Weinberg equilibrium among healthy controls. Statistical analyses were performed using Stata version 14 (StataCorp LP, College Station, TX, USA).

## Results

### Study population

The characteristics of patients and healthy controls are shown in Table 2. The genotype distributions among healthy controls deviated from Hardy-Weinberg equilibrium for *TGF-B1* (rs1800469) (*P =* 0.016), *TLR1* (rs4833095) (*P* = 0.028), *TLR2* (rs4696480) (*P* = 0.018), and *TLR4* (rs1554973) (*P* = 0.032). After correction for multiple testing, none of the deviations remained statistically significant.

### Polymorphisms associated with the risk of PsO in the general population

The association of SNPs in patients with PsO was compared with healthy controls using a dominant model for association. Nine SNPs were nominally associated with PsO (*P* < 0.05) (S3 Table). Two SNPs [*TNF* (rs361525) and *IL12B* (rs6887695)] withstood correction for multiple testing (Table 3).

**Table 3:** Risk estimates for carriers of the variant allele compared to homozygous carriers of the wild type allele.

Gene	Rs number	MAF (cases)	MAF (controls)		Odds ratio (95% CI)	*P*-value	q-value	Effect of minor allele
**PsO compared to healthy controls**								
**IL12B**								
G/C	rs6887695	0.23	0.29	0.68 (0.54–0.85)		0.0010	0.047	Unknown [64] [65]
**TNF**								
G/A	rs361525	0.13	0.04	3.57 (2.49–5.11)		< 1.0 x 10^−6^	0.00011	Decreased expression [66]
**PsC10 compared to healthy controls**								
**TNF**								
G/A	rs361525	0.14	0.04	3.81 (2.45–5.98)		< 1.0 x 10^−6^	0.00011	Decreased expression [66]
**PsA compared to healthy controls**								
**TNF**								
G/A	rs361525	0.09	0.04	2.24 (1.57–3.20)		9.0 x 10^−6^	0.00064	Decreased expression [66]

**Abbreviations:** MAF, minor allele frequencies; PsO, psoriasis; PsA, psoriatic arthritis; PsC, cutaneous psoriasis; PsC10, patients with PsC followed for ≥10 years; CI, confidence interval.

### Polymorphisms associated with the risk of PsC10 in the general population

Patients were stratified for phenotype and diseased duration, and the genotype distributions of patients with PsC10 were compared with healthy controls. Five SNPs were nominally associated with PsC10 (S3 Table), among which one SNP [*TNF* (rs361525)] withstood correction for multiple testing (Table 3).

### Polymorphisms associated with the risk of PsA in the general population

Genotype distributions for patients with PsA from DANBIO were compared with those from healthy controls using a dominant model for association. Two SNPs were nominally associated with PsA (S3 Table), with one withstanding correction for multiple testing (Table 3).

### Polymorphisms associated with the risk of PsA in patients with PsO

Genotype distributions for patients with PsA from DANBIO were compared with patients with PsC10. Only *TNF* (rs361525) (OR: 0.59, 95% CI: 0.38–0.92, *P =* 0.019, q = 0.32) showed nominally evidence of association, but this did not withstand correction for multiple testing (S3 Table).

## Discussion

This study evaluated 53 SNPs in 37 genes in 480 Danish patients with moderate-to-severe PsO and in 459 patients with PsA. The polymorphisms were primarily chosen as functional polymorphisms targeting the inflammatory signaling pathways. Eleven polymorphisms in 10 genes were nominally associated with PsO and/or PsC and/or PsA (*P* < 0.05), among which two withstood correction for multiple testing.

The variant alleles in *IL12B* (rs6887695) [38–45], *IL23R* (rs11209026) [39–42, 46, 47], and *TNF* (rs361525) [48–52] are all well-known polymorphism associated with susceptibility to PsO. We found that the variant allele of *IL23R* (rs11209026) was nominally associated with decreased risk of PsO, but this did not withstand correction for multiple testing.

In the current study, the variant allele of *TNF* (rs361525) was the only variant consistently associated with an increased risk of all phenotypes, thus underlining the importance of TNF-α signaling in the development of psoriasis. Conflicting results for the association of *TNF* (rs361525) and development of PsA in PsO have previously been reported. While some studies have reported a protective role for the variant allele of *TNF* (rs361525) in development of PsA in PsO [53, 54], other studies have reported increased or no altered risk for development of PsA in PsO [55, 56]. In the current study, the variant allele of *TNF* (rs361525) was associated with an increased risk of PsA and nominally associated with a decreased risk of developing PsA in PsO. The strong association between PsO and *TNF* (rs361525), and the increased risk of PsC compared to PsA have been suggested to be due to a high LD between *TNF* (rs361525) and HLA-CW*6 [54].

Another SNP investigated, *IL12B* (rs3212217), is in close LD with two other polymorphisms: *IL12B* (rs3212227, r^2^ = 0.95) [38–45, 57] and *IL12B* (rs2082412, r^2^ = 0.95) [11, 17], both associated with reduced risk of PsO. Differences in effect size between PsC and PsA for *IL12B* (rs2082412) have been reported [11, 17] although the associations could not be replicated by a large GWAS that attempted to confirm previously reported associations [12]. In the current study, no statistically significant difference in effect size between PsA and PsC10 for *IL12B* (rs3212217) was observed. Thus the current study adds to the evidence that suggest that there is no difference in PsA and PsC10 susceptibility for the variants in *IL12B*.

In a previous study, the variant allele of *PPARG* (rs1801282) was associated with decreased risk of PsA [58]. Another study demonstrated the same trend, although the findings were not statistically significant [59]. In the current study, the variant allele of *PPARG* (rs1801282) was nominally associated with decreased risk of PsA, with odds ratios similar to those previously reported, but the association was not statistically significant after correction for multiple testing. In accordance with previous studies [60], we observed no association between *PPARG* (rs1801282) and uncomplicated psoriasis, PsO or PsC10. Additional studies investigating association of *PPARG* (rs1801282) with PsA should be performed in order to clarify the role of *PPARG* (rs1801282) in PsA. *PPARG* (rs1801282) encodes a Pro to Ala amino acid substitution in *PPARG* that leads to reduced activity. *PPARG* (rs1801282) is a variant of notable interest that has, in addition to PsA, been associated with a lower risk of alcohol-related breast cancer [61, 62], but an increased risk of alcohol-related colorectal cancer [63] likely because *PPARG* inhibits sex hormone synthesis via negative regulation of aromatase [62].

We failed to replicate *PTPN22* (rs2476601) as a risk factor of PsA in PsO, probably due to lack of power, since we observed odds ratios similar to those previous reported [12, 20].

The main limitation in this and other studies evaluating risk of PsA in PsO is the potential for phenotype misclassification, which inevitably will lead to a decrease in statistical power. In this study, data on PsA, for the DERMBIO cohort, were retrieved from the DERMBIO database, with patient registration performed by dermatologists. Although dermatologist are experienced and trained in assessing PsA, we cannot rule out the possibility that some patients with PsC may have had undiagnosed or undeveloped PsA. This might bias the effect size towards the null hypothesis of no difference. In order to overcome the possibility of undeveloped PsA and reduce the risk of undiagnosed PsA, a cohort of patients with PsC who had the disease for ≥10 years (PsC10) was established, as proposed by Stuart et al [12]. Similarly, there is risk of potential misclassification in the PsA cohort (e.g. rheumatoid arthritis or osteoarthritis). However, this risk was lowered by only including patients registered in DANBIO, where diagnoses are according to the treating rheumatologist.

In conclusion, this study confirms that two SNPs in the *IL12B* and *TNF* genes are associated with susceptibility to psoriasis in Danish patients with moderate-to-severe psoriasis. None of the investigated SNPs were specifically associated with isolated cutaneous psoriasis or psoriatic arthritis.

## Supporting information

S1 Table The chosen polymorphisms and corresponding gene. (DOCX)

S2 Table Genotype distributions for patients with psoriasis, psoriatic arthritis, isolated cutaneous psoriasis for a minimum of 10 years, and healthy controls. (DOCX)

S3 Table Odds ratios (OR) for genotypes studied among healthy controls and patients with psoriasis (PsO), isolated cutaneous psoriasis (PsC10) and psoriatic arthritis (PsA) and comparison of PsA with PsC10, unadjusted. (DOCX)

S4 Table Odds ratios (OR) for genotypes studied among healthy controls and patients with psoriasis (PsO), isolated cutaneous psoriasis (PsC10) and psoriatic arthritis (PsA) and comparison of PsA with PsC10 adjusted for age and gender. (DOCX)
