# Supplementary material for: Genetic polymorphisms associated with psoriasis and development of psoriatic arthritis in patients with psoriasis
Source: PLoS One. 2018 Feb 1;13(2):e0192010. doi: 10.1371/journal.pone.0192010 (PMC5794107; doi:10.1371/journal.pone.0192010)
Supplement: S1 Table — (DOCX) [file pone.0192010.s001.docx]

| **Supplementary Table 1. Chosen polymorphisms and corresponding gene.** Associated effect of polymorphism. | | | | | |
| --- | --- | --- | --- | --- | --- |
| GENE | GENETIC VARIANT | WILDTYPE/  VARIANT | NCBI GENE ID | PROTEIN FUNCTION | EFFECT OF VARIANT ALLELE |
| *CARD8* | rs2043211 | A/T | 22900 | CARD8 is part of the development of the inflammasome. | Decreased expression [1] and associated with worse disease course in early rheumatoid arthritis (RA) [2]. |
| *CD14* | rs2569190 | G/A | 929 | CD14 binds LPS and transport it to TLR4 | Homozygote variant increases CD14 level [3, 4]. |
| *IFNG* | rs2430561 | T/A | 3458 | IFN-γ is a pro- and anti-inflammatory cytokine. Increased levels of IFN-γ in synovial fluid IFN-γ is observed in patients with psoriatic arthritis (PsA) [5]. | Increased IFN-γ level [6, 7]. |
| *IFNGR1* | rs2234711 | T/C | 3459 | IFN-y receptor is a heterodimer of IFNGR1 and IFNGR2. Ligand binding activates JAK-STAT pathway. | Increased expression of IFNGR1 [8, 9]. |
| *IFNGR2* | rs17882748 | C/T | 3460 |  | C increases expression in haplotype with rs8126756T [10]. |
| *IFNGR2* | rs8126756 | T/C | 3460 |  | T increases expression in haplotype with rs17882748C [10]. |
| *IL1B* | rs1143623 | G/C | 3553 | IL-1β is activated by NFKB1 and works as a pro-inflammatory cytokine. | Decreased IL-1β level [11] but is only found in haplotypes with increased IL-1β transcription [12]. |
| *IL1B* | rs1143627 | T/C | 3553 |  | Decreased IL-1β expression [11, 13] but is only seen in haplotypes with increased IL-1β transcription [12]. |
| *IL1B* | rs4848306 | G/A | 3553 |  | Decreased IL-1β transcription [11-13]. |
| *IL1RN* | rs4251961 | T/C | 3557 | IL-1RA works by binding to the IL-1 receptor and thereby inhibiting IL-1β signalling. | Decreased IL-1RA level [14, 15]. |
| *IL4R* | rs1805010 | A/G | 3566 | IL-4 binds to the IL-4 receptor and significantly inhibits IL-17 production | Increased IL-17 level [16]. |
| *IL6* | rs10499563 | T/C | 3569 | IL-6 is activated by NFKB1 and works as a pro- and anti-inflammatory cytokine. | Decreased expression of IL-6 [17]. |
| *IL6R* | rs4537545 | C/T | 3570 | IL-6 binds to the IL-6 receptor and initiates a kinase cascade. | Homozygote variant increases IL-6r and IL-6 levels [18]. |
| *IL10* | rs1800872 | C/A | 3586 | IL-10 is a regulatory cytokine capable of inhibiting pro-inflammatory cytokines such as IFN-γ and TNF-α. IL-10 is activated by NFKB1. | Increased IL-10 expression [19]. |
| *IL10* | rs3024505 | C/T | 3586 |  | Unknown function. Associated with Cohn’s disease (CD) and ulcerative colitis (UC) [20-22]. |
| *IL12B* | rs3212217 | G/C | 3593 | IL-12-p40 (B) serves as a subunit of interleukin 12 and 23. | Increased IL12B level [23]. |
| *IL12B* | rs6887695 | G/C | 3593 |  | Unknown [24] [25]. Associated with psoriasis [26] and CD [27]. |
| *IL12RB1* | rs401502 | C/G | 3594 | IL12RB1 work as a shared subunit of IL-12 receptor and IL-23. | Reduced IL-12p40 level [28]. |
| *IL17A* | rs2275913 | G/A | 3605 | IL-17A is a pro-inflammatory cytokine and a potent mediator in delayed type reactions. | Increased IL-17A expression [29]. |
| *IL18* | rs187238 | G/C | 3606 | IL-18 is a pro-inflammatory cytokine, which feedback activates IFN-γ. IL-18 is known to enhance the production of IL-17, TNF-α, and IL-1β. | Reduced IL-18 level [30] and expression [31]. |
| *IL18* | rs1946518 | G/T | 3606 |  | TT reduces IL-18 level [30, 32] and expression [31].  Haplotype with rs1946518G and rs187238G increases transcription [33, 34]. |
| *IL23R* | rs11209026 | G/A | 14233 | IL-23 binds to the IL-23 receptor and induces production of IL-17. | Decreased production of IL-17 [35] and is associated with psoriasis [26], CD [36], and UC [20]. |
| *JAK2* | rs12343867 | T/C | 3717 | JAK2 is a kinase, which interacts with numerous membrane receptors including IFNGR, IL12R, and IL23R. | Reduced expression of JAK2 [37]. |
| *LY96 (MD2)* | rs11465996 | C/G | 23643 | MD2 is involved in binding of LPS to the TLR4 complex. | Increased LY96 (MD2) and TNF-α levels [38]. |
| *MAP3K14 (NIK)* | rs7222094 | T/C | 9020 | NIK binds to TRAF2 and work as a central kinase in the non-canonical NFKB pathway. | Homozygote variant decreases CXCL10 protein levels [39]. |
| *NFKB1* | rs28362491 | ins/del | 4790 | NFκB1 (p50) is a transcription factor. The p50 subunit of NFκB can both act pro-inflammatory as part of the p50/p65 complex and as p50 homodimer it can act anti-inflammatory [40]. | Del decreases p50 subunit expression [41]. |
| *NFKBIA* | rs696 | G/A | 4792 | NFκBIα (IκBα) is an inhibitor of NFκB1 | Increased NFκBIα expression [42]. |
| *NLRP1* | rs2670660 | A/G | 22861 | NLRP1 is part of the NLRP1 inflammasome and is central in innate immunity and inflammation. Activation of the inflammasome leads to processing and release of IL-1β and IL-18. | Reduced NLRP1 transcription [43]. |
| *NLRP1* | rs878329 | G/C | 22861 |  | C reduced mRNA levels  GG associated with RA [44]. |
| *NLRP3* | rs10754558 | C/G | 114548 | NLRP3 is part of the NLRP3 inflammasome complex and is central in innate immunity and inflammation. Activation of the inflammasome leads to processing and release of IL-1β and IL-18. | Increased expression and mRNA stability [45]. |
| *NLRP3* | rs4612666 | C/T | 114548 |  | Decreased expression [46]. |
| *PPARG* | rs1801282 | C/G | 5468 | PPARγ is a transcription factor | Amino acid substitution and decreased PPARg mRNA level, but increased MyD88, TLR4, TLR5, TLR9, P65 and TNF-α mRNA levels [47].  C is associated with PsA [48]. |
| *PTPN22* | rs2476601 | G/A | 26191 | PTPN22 is involved in regulation of multiple signalling pathways associated with inflammation. | A decreases TNF-α level in serum [49].  G associated with CD [36]. |
| *SUMO4* | rs237025 | T/C | 387082 | SUMO4 modifies IκBα, which leads to negative regulation of NFκB transcriptional activity. | Increased NFκB1 expression [50]. |
| *TBX21* | rs17250932 | T/C | 30009 | TBX21 works as a transcription factor that controls IFN-γ expression. | Reduced TBX21 transcription. Reduced T-bet (TBX21) and IFN-γ level and increased IL-4 level [51]. |
| *TGFB1* | rs1800469 | C/T | 7040 | TGF-β1 is a regulatory cytokine which can inhibit the secretion and activity of many other cytokines including IFN-γ and TNF-α. | Increased expression [52]. |
| *TIRAP* | rs8177374 | C/T | 114609 | TIRAP is an adapter molecule which is involved in TLR4 signalling. | Increased TNF-α, IL-6, and IFN-γ levels [53]. |
| *TLR1* | rs4833095 | T/C | 7096 | TLRs are important in pathogen recognition and activation of innate immunity and activate inflammation through the canonical NFKB pathway. | Increased TLR1 level [54]. |
| *TLR2* | rs11938228 | C/A | 7097 | See above | Unknown [55] |
| *TLR2* | rs1816702 | C/T | 7097 | See above | Increased receptor level [56] |
| *TLR2* | rs3804099 | T/C | 7097 | See above | Decreased TNF-α, IL-1β, and IL-6 levels [57] |
| *TLR2* | rs4696480 | A/T | 7097 | See above | Unknown [55] |
| *TLR4* | rs12377632 | T/C | 7099 | See above | Unknown [55] |
| *TLR4* | rs1554973 | T/C | 7099 | See above | Unknown [55] |
| *TLR4* | rs5030728 | G/A | 7099 | See above | Unknown [55] |
| *TLR5* | rs5744168 | C/T | 7100 | See above | Decreased TNF-α, IL-1β, IL-6 levels [57] and inhibited TLR5 function [58]. |
| *TLR5* | rs5744174 | T/C | 7100 | See above | Decreased mRNA levels of IL-6 & IL-1β [59] increased production of CCL20 [60] and IFN-γ level [61] |
| *TLR9* | rs187084 | T/C | 54106 | See above | Decreased expression of TLR9 [62] |
| *TLR9* | rs352139 | G/A | 54106 | See above | Increased expression of TLR9 [62] |
| *TNF* | rs1800629 | G/A | 7124 | TNF-α is activated by NFKB1 and works as a pro-inflammatory cytokine. | Increased expression [63] |
| *TNF* | rs361525 | G/A | 7124 |  | Decreased expression [64], associated with psoriasis and PsA [65] |
| *TNFAIP3* | rs6927172 | C/G | 7128 | TNFAIP3 (A20) inhibits NFκB activation and TNF-α mediated apoptosis. | Increased expression [66] |
| *TNFRSF1A* | rs4149570 | G/T | 7132 | TNF-α binds to TNF receptor 1 (TNFR1) which leads to a kinase cascade initiation. | Increased expression [67] |

1. Paramel GV, Folkersen L, Strawbridge RJ, Elmabsout AA, Särndahl E, Lundman P, et al. CARD8 gene encoding a protein of innate immunity is expressed in human atherosclerosis and associated with markers of inflammation. Clinical Science. 2013;125(8):401-7.

2. Kastbom A, Johansson M, Verma D, Söderkvist P, Rantapää-Dahlqvist S. CARD8 p. C10X polymorphism is associated with inflammatory activity in early rheumatoid arthritis. Annals of the rheumatic diseases. 2010;69(4):723-6.

3. Mertens J, Bregadze R, Mansur A, Askar E, Bickeböller H, Ramadori G, et al. Functional impact of endotoxin receptor CD14 polymorphisms on transcriptional activity. Journal of molecular medicine. 2009;87(8):815-24.

4. Baldini M, Carla Lohman I, Halonen M, Erickson RP, Holt PG, Martinez FD. A Polymorphism* in the 5′ flanking region of the CD14 gene is associated with circulating soluble CD14 levels and with total serum immunoglobulin E. American journal of respiratory cell and molecular biology. 1999;20(5):976-83.

5. Muntyanu A, Abji F, Liang K, Pollock RA, Chandran V, Gladman DD. Differential gene and protein expression of chemokines and cytokines in synovial fluid of patients with arthritis. Arthritis Res Ther. 2016;18(1):296. Epub 2016/12/15. doi: 10.1186/s13075-016-1196-6. PubMed PMID: 27964744; PubMed Central PMCID: PMCPMC5154157.

6. Schena FP, Cerullo G, Torres DD, Scolari F, Foramitti M, Amoroso A, et al. Role of interferon-γ gene polymorphisms in susceptibility to IgA nephropathy: a family-based association study. European journal of human genetics. 2006;14(4):488-96.

7. Pravica V, Perrey C, Stevens A, Lee J-H, Hutchinson IV. A single nucleotide polymorphism in the first intron of the human IFN-γ gene:: Absolute correlation with a polymorphic CA microsatellite marker of high IFN-γ production. Human immunology. 2000;61(9):863-6.

8. Canedo P, Corso G, Pereira F, Lunet N, Suriano G, Figueiredo C, et al. The interferon gamma receptor 1 (IFNGR1)− 56C/T gene polymorphism is associated with increased risk of early gastric carcinoma. Gut. 2008;57(11):1504-8.

9. Matsuda A, Ebihara N, Kumagai N, Fukuda K, Ebe K, Hirano K, et al. Genetic polymorphisms in the promoter of the interferon gamma receptor 1 gene are associated with atopic cataracts. Investigative ophthalmology & visual science. 2007;48(2):583-9.

10. Hijikata M, Shojima J, Matsushita I, Tokunaga K, Ohashi J, Hang NT, et al. Association of IFNGR2 gene polymorphisms with pulmonary tuberculosis among the Vietnamese. Human genetics. 2012;131(5):675-82.

11. Wen A-Q, Gu W, Wang J, Feng K, Qin L, Ying C, et al. Clinical relevance of IL-1β promoter polymorphisms (− 1470,− 511, and− 31) in patients with major trauma. Shock. 2010;33(6):576-82.

12. Chen H, Wilkins LM, Aziz N, Cannings C, Wyllie DH, Bingle C, et al. Single nucleotide polymorphisms in the human interleukin-1B gene affect transcription according to haplotype context. Human molecular genetics. 2006;15(4):519-29.

13. Lind H, Haugen A, Zienolddiny S. Differential binding of proteins to the IL1B− 31 T/C polymorphism in lung epithelial cells. Cytokine. 2007;38(1):43-8.

14. Rafiq S, Stevens K, Hurst A, Murray A, Henley W, Weedon M, et al. Common genetic variation in the gene encoding interleukin-1-receptor antagonist (IL-1RA) is associated with altered circulating IL-1RA levels. Genes and immunity. 2007;8(4):344-51.

15. Carrol ED, Payton A, Payne D, Miyajima F, Chaponda M, Mankhambo LA, et al. The IL1RN promoter rs4251961 correlates with IL-1 receptor antagonist concentrations in human infection and is differentially regulated by GATA-1. The Journal of Immunology. 2011;186(4):2329-35.

16. Wallis SK, Cooney LA, Endres JL, Lee MJ, Ryu J, Somers EC, et al. A polymorphism in the interleukin-4 receptor affects the ability of interleukin-4 to regulate Th17 cells: a possible immunoregulatory mechanism for genetic control of the severity of rheumatoid arthritis. Arthritis research & therapy. 2011;13(1):R15.

17. Smith AJ, D’Aiuto F, Palmen J, Cooper JA, Samuel J, Thompson S, et al. Association of serum interleukin-6 concentration with a functional IL6− 6331T> C polymorphism. Clinical chemistry. 2008;54(5):841-50.

18. Rafiq S, Frayling T, Murray A, Hurst A, Stevens K, Weedon M, et al. A common variant of the interleukin 6 receptor (IL-6r) gene increases IL-6r and IL-6 levels, without other inflammatory effects. Genes and immunity. 2007;8(7):552-9.

19. Rees L, Wood N, Gillespie K, Lai K, Gaston K, Mathieson P. The interleukin-10-1082 G/A polymorphism: allele frequency in different populations and functional significance. Cellular and molecular life sciences. 2002;59(3):560-9.

20. Anderson CA, Boucher G, Lees CW, Franke A, D'Amato M, Taylor KD, et al. Meta-analysis identifies 29 additional ulcerative colitis risk loci, increasing the number of confirmed associations to 47. Nature genetics. 2011;43(3):246-52.

21. Andersen V, Ernst A, Christensen J, Østergaard M, Jacobsen BA, Tjønneland A, et al. The polymorphism rs3024505 proximal to IL-10 is associated with risk of ulcerative colitis and Crohns disease in a Danish case-control study. BMC medical genetics. 2010;11(1):82.

22. Bank S, Andersen PS, Burisch J, Pedersen N, Roug S, Galsgaard J, et al. Polymorphisms in the inflammatory pathway genes TLR2, TLR4, TLR9, LY96, NFKBIA, NFKB1, TNFA, TNFRSF1A, IL6R, IL10, IL23R, PTPN22, and PPARG are associated with susceptibility of inflammatory bowel disease in a Danish cohort. PloS one. 2014;9(6):e98815.

23. Wu JF, Wu TC, Chen CH, Ni YH, Chen HL, Hsu HY, et al. Serum levels of interleukin-10 and interleukin-12 predict early, spontaneous hepatitis B virus e antigen seroconversion. Gastroenterology. 2010;138(1):165-72 e1-3. Epub 2009/09/29. doi: 10.1053/j.gastro.2009.09.018. PubMed PMID: 19782084.

24. Eskandari-Nasab E, Moghadampour M, Asadi-Saghandi A, Kharazi-Nejad E, Rezaeifar A, Pourmasoumi H. Levels of interleukin-(IL)-12p40 are markedly increased in Brucellosis among patients with specific IL-12B genotypes. Scand J Immunol. 2013;78(1):85-91. Epub 2013/04/13. doi: 10.1111/sji.12054. PubMed PMID: 23578145.

25. Wang X, Wu T, Zhou F, Liu S, Zhou R, Zhu S, et al. IL12p40 regulates functional development of human CD4+ T cells: enlightenment by the elevated expressions of IL12p40 in patients with inflammatory bowel diseases. Medicine (Baltimore). 2015;94(10):e613. Epub 2015/03/12. doi: 10.1097/md.0000000000000613. PubMed PMID: 25761185; PubMed Central PMCID: PMCPMC4602478.

26. Lee YH, Song GG. Associations between interleukin-23R and interleukin-12B polymorphisms and psoriasis susceptibility: a meta-analysis. Immunological investigations. 2013;42(8):726-36.

27. Parkes M, Barrett JC, Prescott NJ, Tremelling M, Anderson CA, Fisher SA, et al. Sequence variants in the autophagy gene IRGM and multiple other replicating loci contribute to Crohn's disease susceptibility. Nature genetics. 2007;39(7):ng2061.

28. Tao Y-P, Wang W-L, Li S-Y, Zhang J, Shi Q-Z, Zhao F, et al. Associations between polymorphisms in IL-12A, IL-12B, IL-12Rβ1, IL-27 gene and serum levels of IL-12p40, IL-27p28 with esophageal cancer. Journal of cancer research and clinical oncology. 2012;138(11):1891-900.

29. Espinoza JL, Takami A, Nakata K, Onizuka M, Kawase T, Akiyama H, et al. A genetic variant in the IL-17 promoter is functionally associated with acute graft-versus-host disease after unrelated bone marrow transplantation. PloS one. 2011;6(10):e26229.

30. Jaiswal PK, Singh V, Srivastava P, Mittal RD. Association of IL-12, IL-18 variants and serum IL-18 with bladder cancer susceptibility in North Indian population. Gene. 2013;519(1):128-34.

31. Dziedziejko V, Kurzawski M, Paczkowska E, Machalinski B, Pawlik A. The impact of IL18 gene polymorphisms on mRNA levels and interleukin-18 release by peripheral blood mononuclear cells. Advances in Hygiene & Experimental Medicine/Postepy Higieny i Medycyny Doswiadczalnej. 2012;66.

32. Chen D-Y, Chen Y-M, Chen H-H, Hsieh C-W, Lin C-C, Lan J-L. Functional association of interleukin 18 gene− 607 (C/A) promoter polymorphisms with disease course in Chinese patients with adult-onset Still’s disease. The Journal of rheumatology. 2009;36(10):2284-9.

33. Giedraitis V, He B, Huang W-X, Hillert J. Cloning and mutation analysis of the human IL-18 promoter: a possible role of polymorphisms in expression regulation. Journal of neuroimmunology. 2001;112(1):146-52.

34. Kim SH, Son J, Yang EM, Kim JE, Park HS. A functional promoter polymorphism of the human IL18 gene is associated with aspirin‐induced urticaria. British Journal of Dermatology. 2011;165(5):976-84.

35. Oosting M, ter Hofstede H, van de Veerdonk FL, Sturm P, Kullberg B-J, van der Meer JW, et al. Role of interleukin-23 (IL-23) receptor signaling for IL-17 responses in human Lyme disease. Infection and immunity. 2011;79(11):4681-7.

36. Franke A, McGovern DP, Barrett JC, Wang K, Radford-Smith GL, Ahmad T, et al. Genome-wide meta-analysis increases to 71 the number of confirmed Crohn's disease susceptibility loci. Nature genetics. 2010;42(12):1118-25.

37. Spasovski V, Tosic N, Nikcevic G, Stojiljkovic M, Zukic B, Radmilovic M, et al. The influence of novel transcriptional regulatory element in intron 14 on the expression of Janus kinase 2 gene in myeloproliferative neoplasms. Journal of applied genetics. 2013;54(1):21-6.

38. Gu W, Shan Y-a, Zhou J, Jiang D-p, Zhang L, Du D-y, et al. Functional significance of gene polymorphisms in the promoter of myeloid differentiation-2. Annals of surgery. 2007;246(1):151.

39. Thair SA, Walley KR, Nakada T-a, McConechy MK, Boyd JH, Wellman H, et al. A single nucleotide polymorphism in NF-κB inducing kinase is associated with mortality in septic shock. The Journal of Immunology. 2011;186(4):2321-8.

40. Vogel U, Jensen MK, Due KM, Rimm EB, Wallin H, Nielsen MR, et al. The NFKB1 ATTG ins/del polymorphism and risk of coronary heart disease in three independent populations. Atherosclerosis. 2011;219(1):200-4.

41. Park J-Y, Farrance IK, Fenty NM, Hagberg JM, Roth SM, Mosser DM, et al. NFKB1 promoter variation implicates shear-induced NOS3 gene expression and endothelial function in prehypertensives and stage I hypertensives. American Journal of Physiology-Heart and Circulatory Physiology. 2007;293(4):H2320-H7.

42. Song S, Chen D, Lu J, Liao J, Luo Y, Yang Z, et al. NFκB1 and NFκBIA polymorphisms are associated with increased risk for sporadic colorectal cancer in a southern Chinese population. PloS one. 2011;6(6):e21726.

43. Glinskii AB, Ma J, Ma S, Grant D, Lim C-U, Sell S, et al. Identification of intergenic trans-regulatory RNAs containing a disease-linked SNP sequence and targeting cell cycle progression/differentiation pathways in multiple common human disorders. Cell Cycle. 2009;8(23):3925-42.

44. Sui J, Li H, Fang Y, Liu Y, Li M, Zhong B, et al. NLRP1 gene polymorphism influences gene transcription and is a risk factor for rheumatoid arthritis in han chinese. Arthritis & Rheumatology. 2012;64(3):647-54.

45. Muise AM, Walters T, Xu W, Shen–Tu G, Guo CH, Fattouh R, et al. Single nucleotide polymorphisms that increase expression of the guanosine triphosphatase RAC1 are associated with ulcerative colitis. Gastroenterology. 2011;141(2):633-41.

46. Hitomi Y, Ebisawa M, Tomikawa M, Imai T, Komata T, Hirota T, et al. Associations of functional NLRP3 polymorphisms with susceptibility to food-induced anaphylaxis and aspirin-induced asthma. Journal of Allergy and Clinical Immunology. 2009;124(4):779-85. e6.

47. Aoyagi Y, Nagata S, Kudo T, Fujii T, Wada M, Chiba Y, et al. Peroxisome proliferator-activated receptor gamma 2 mutation may cause a subset of ulcerative colitis. Pediatr Int. 2010;52(5):729-34. Epub 2010/07/02. doi: 10.1111/j.1442-200X.2010.03195.x. PubMed PMID: 20591056.

48. Butt C, Gladman D, Rahman P. PPAR-gamma gene polymorphisms and psoriatic arthritis. J Rheumatol. 2006;33(8):1631-3. Epub 2006/06/20. PubMed PMID: 16783862.

49. Kariuki SN, Crow MK, Niewold TB. The PTPN22 C1858T polymorphism is associated with skewing of cytokine profiles toward high interferon‐α activity and low tumor necrosis factor α levels in patients with lupus. Arthritis & Rheumatology. 2008;58(9):2818-23.

50. Guo D, Li M, Zhang Y, Yang P, Eckenrode S, Hopkins D, et al. A functional variant of SUMO4, a new IκBα modifier, is associated with type 1 diabetes. Nature genetics. 2004;36(8):837-41.

51. Li J, Li J, You Y, Chen S. The role of upstream stimulatory factor 1 in the transcriptional regulation of the human TBX21 promoter mediated by the T-1514C polymorphism associated with systemic lupus erythematosus. Immunogenetics. 2012;64(5):361-70.

52. Shah R, Hurley CK, Posch PE. A molecular mechanism for the differential regulation of TGF-β1 expression due to the common SNP− 509C-T (c.− 1347C> T). Human genetics. 2006;120(4):461-9.

53. Ferwerda B, Alonso S, Banahan K, McCall MB, Giamarellos-Bourboulis EJ, Ramakers BP, et al. Functional and genetic evidence that the Mal/TIRAP allele variant 180L has been selected by providing protection against septic shock. Proceedings of the National Academy of Sciences. 2009;106(25):10272-7.

54. Uciechowski P, Imhoff H, Lange C, Meyer CG, Browne EN, Kirsten DK, et al. Susceptibility to tuberculosis is associated with TLR1 polymorphisms resulting in a lack of TLR1 cell surface expression. Journal of leukocyte biology. 2011;90(2):377-88.

55. Gast A, Bermejo JL, Claus R, Brandt A, Weires M, Weber A, et al. Association of inherited variation in Toll-like receptor genes with malignant melanoma susceptibility and survival. PLoS One. 2011;6(9):e24370.

56. Bielinski SJ, Hall JL, Pankow JS, Boerwinkle E, Matijevic-Aleksic N, He M, et al. Genetic variants in TLR2 and TLR4 are associated with markers of monocyte activation: the Atherosclerosis Risk in Communities MRI Study. Human genetics. 2011;129(6):655-62.

57. Zhang F, Gao X-D, Wu W-W, Gao Y, Zhang Y-W, Wang S-P. Polymorphisms in toll-like receptors 2, 4 and 5 are associated with Legionella pneumophila infection. Infection. 2013;41(5):941-8.

58. Hawn TR, Verbon A, Lettinga KD, Zhao LP, Li SS, Laws RJ, et al. A common dominant TLR5 stop codon polymorphism abolishes flagellin signaling and is associated with susceptibility to legionnaires' disease. Journal of Experimental Medicine. 2003;198(10):1563-72.

59. Klimosch SN, Försti A, Eckert J, Knežević J, Bevier M, von Schönfels W, et al. Functional TLR5 Genetic Variants Affect Human Colorectal Cancer Survival. Cancer Research. 2013;73(24):7232.

60. Sheridan J, Mack DR, Amre DK, Israel DM, Cherkasov A, Li H, et al. A Non-Synonymous Coding Variant (L616F) in the TLR5 Gene Is Potentially Associated with Crohn's Disease and Influences Responses to Bacterial Flagellin. PLoS ONE. 2013;8(4):e61326. doi: 10.1371/journal.pone.0061326. PubMed PMID: PMC3623901.

61. Dhiman N, Ovsyannikova IG, Vierkant RA, Ryan JE, Pankratz VS, Jacobson RM, et al. Associations Between SNPs in Toll-like Receptors and Related Intracellular Signaling Molecules and Immune Responses to Measles Vaccine: Preliminary Results. Vaccine. 2008;26(14):1731-6. doi: 10.1016/j.vaccine.2008.01.017. PubMed PMID: PMC2292110.

62. Tao K, Fujii M, Tsukumo Si, Maekawa Y, Kishihara K, Kimoto Y, et al. Genetic variations of Toll‐like receptor 9 predispose to systemic lupus erythematosus in Japanese population. Annals of the Rheumatic Diseases. 2007;66(7):905-9. doi: 10.1136/ard.2006.065961. PubMed PMID: PMC1955115.

63. Karimi M, Goldie LC, Cruickshank MN, Moses EK, Abraham LJ. A critical assessment of the factors affecting reporter gene assays for promoter SNP function: a reassessment of− 308 TNF polymorphism function using a novel integrated reporter system. European journal of human genetics. 2009;17(11):1454-62.

64. Kaluza W, Reuss E, Grossmann S, Hug R, Schopf RE, Galle PR, et al. Different transcriptional activity and in vitro TNF-alpha production in psoriasis patients carrying the TNF-alpha 238A promoter polymorphism. J Invest Dermatol. 2000;114(6):1180-3. Epub 2000/06/09. doi: 10.1046/j.1523-1747.2000.00001.x. PubMed PMID: 10844563.

65. Jia Y, Qin H, Zhang J, Liu X, Li L. Association of the tumour necrosis factor‐α polymorphisms rs361525 and rs1800629 with susceptibility to psoriasis: a meta‐analysis. Clinical and experimental dermatology. 2013;38(8):836-44.

66. Elsby LM, Orozco G, Denton J, Worthington J, Ray DW, Donn RP. Functional evaluation of TNFAIP3 (A20) in rheumatoid arthritis. Clinical and experimental rheumatology. 2010;28(5):708.

67. Wang G-B, Li C-R, Yang J, Wen P-Q, Jia S-L. A regulatory polymorphism in promoter region of TNFR1 gene is associated with Kawasaki disease in Chinese individuals. Human immunology. 2011;72(5):451-7.
