# Supplementary material for: Genetic polymorphisms associated with psoriasis and development of psoriatic arthritis in patients with psoriasis
Source: PLoS One. 2018 Feb 1;13(2):e0192010. doi: 10.1371/journal.pone.0192010 (PMC5794107; doi:10.1371/journal.pone.0192010)
Supplement: S2 Table — (DOCX) [file pone.0192010.s002.docx]

| **Supplementary Table 2.** Genotype distributions for patients with psoriasis, psoriatic arthritis, isolated cutaneous psoriasis for ≥10 years, and healthy controls. | | | | | |
| --- | --- | --- | --- | --- | --- |
|  |  | **PsO** | **PsC10** | **PsA** | **Controls** |
| *CARD8* (rs2043211) | |  |  |  |  |
| AA |  | 228 | 69 | 201 | 321 |
| AT |  | 204 | 72 | 217 | 342 |
| TT |  | 46 | 10 | 39 | 94 |
| *CD14* (rs2569190) |  |  |  |  |  |
| GG |  | 125 | 36 | 130 | 236 |
| GA |  | 229 | 73 | 233 | 360 |
| AA |  | 119 | 41 | 94 | 170 |
| *IFNG* (rs2430561) |  |  |  |  |  |
| TT |  | 131 | 47 | 138 | 199 |
| TA |  | 240 | 70 | 216 | 398 |
| AA |  | 99 | 31 | 102 | 161 |
| *IFNGR1* (rs2234711) | |  |  |  |  |
| TT |  | 168 | 66 | 173 | 290 |
| TC |  | 213 | 61 | 218 | 361 |
| CC |  | 91 | 24 | 63 | 119 |
| *IFNGR2* (rs17882748) | |  |  |  |  |
| CC |  | 135 | 45 | 119 | 199 |
| CT |  | 223 | 67 | 233 | 391 |
| TT |  | 113 | 33 | 106 | 153 |
| *IFNGR2* (rs8126756) | |  |  |  |  |
| TT |  | 349 | 100 | 343 | 553 |
| TC |  | 110 | 43 | 104 | 168 |
| CC |  | 15 | 6 | 9 | 18 |
| *IL1B* (rs1143623) |  |  |  |  |  |
| GG |  | 257 | 77 | 229 | 401 |
| GC |  | 179 | 61 | 198 | 316 |
| CC |  | 39 | 11 | 32 | 55 |
| *IL1B* (rs1143627) |  |  |  |  |  |
| TT |  | 214 | 67 | 183 | 340 |
| TC |  | 203 | 65 | 222 | 339 |
| CC |  | 61 | 18 | 53 | 97 |
| *IL1B* (rs4848306) |  |  |  |  |  |
| GG |  | 141 | 38 | 149 | 246 |
| GA |  | 239 | 83 | 232 | 373 |
| AA |  | 97 | 30 | 76 | 151 |
| *IL1RN* (rs4251961) |  |  |  |  |  |
| TT |  | 200 | 64 | 191 | 298 |
| TC |  | 206 | 67 | 201 | 360 |
| CC |  | 71 | 19 | 66 | 112 |
| *IL4R* (rs1805010) |  |  |  |  |  |
| AA |  | 127 | 45 | 139 | 209 |
| AG |  | 245 | 74 | 220 | 410 |
| GG |  | 105 | 31 | 98 | 157 |
| *IL6* (rs10499563) |  |  |  |  |  |
| TT |  | 309 | 98 | 286 | 476 |
| TC |  | 149 | 46 | 150 | 259 |
| CC |  | 17 | 5 | 20 | 35 |
| *IL6R* (rs4537545) |  |  |  |  |  |
| CC |  | 167 | 58 | 168 | 289 |
| CT |  | 229 | 66 | 217 | 369 |
| TT |  | 80 | 24 | 70 | 117 |
| *IL10* (rs1800872) |  |  |  |  |  |
| CC |  | 296 | 87 | 278 | 482 |
| CA |  | 149 | 49 | 151 | 258 |
| AA |  | 32 | 15 | 27 | 35 |
| *IL10* (rs3024505) |  |  |  |  |  |
| CC |  | 321 | 99 | 309 | 518 |
| CT |  | 137 | 46 | 131 | 221 |
| TT |  | 17 | 6 | 16 | 22 |
| *IL12B* (rs3212217) |  |  |  |  |  |
| GG |  | 364 | 110 | 320 | 499 |
| GC |  | 107 | 36 | 121 | 235 |
| CC |  | 8 | 5 | 14 | 25 |
| *IL12B* (rs6887695) |  |  |  |  |  |
| GG |  | 290 | 87 | 243 | 385 |
| GC |  | 158 | 53 | 173 | 293 |
| CC |  | 28 | 10 | 39 | 72 |
| *IL12RB1* (rs401502) | |  |  |  |  |
| CC |  | 227 | 75 | 206 | 360 |
| CG |  | 198 | 55 | 203 | 303 |
| GG |  | 53 | 20 | 47 | 87 |
| *IL17A* (rs2275913) |  |  |  |  |  |
| GG |  | 197 | 62 | 177 | 340 |
| GA |  | 214 | 67 | 217 | 336 |
| AA |  | 67 | 22 | 60 | 95 |
| *IL18* (rs187238) |  |  |  |  |  |
| GG |  | 246 | 76 | 241 | 387 |
| GC |  | 190 | 60 | 176 | 312 |
| CC |  | 41 | 14 | 40 | 64 |
| *IL18* (rs1946518) |  |  |  |  |  |
| GG |  | 185 | 59 | 166 | 282 |
| GT |  | 217 | 67 | 219 | 363 |
| TT |  | 76 | 25 | 72 | 113 |
| *IL23R* (rs11209026) | |  |  |  |  |
| GG |  | 448 | 144 | 419 | 680 |
| GA |  | 31 | 7 | 39 | 89 |
| AA |  | 0 | 0 | 1 | 5 |
| *JAK2* (rs12343867) |  |  |  |  |  |
| TT |  | 251 | 75 | 236 | 398 |
| TC |  | 195 | 65 | 188 | 299 |
| CC |  | 29 | 8 | 33 | 61 |
| *LY96* (rs11465996) |  |  |  |  |  |
| CC |  | 229 | 62 | 207 | 344 |
| CG |  | 204 | 73 | 208 | 337 |
| GG |  | 46 | 16 | 42 | 81 |
| *MAP3k14* (rs7222094) | |  |  |  |  |
| TT |  | 159 | 51 | 141 | 235 |
| TC |  | 239 | 73 | 235 | 383 |
| CC |  | 79 | 27 | 80 | 147 |
| *NFKB1* (rs28362491) | |  |  |  |  |
| I/I |  | 181 | 53 | 166 | 269 |
| I/D |  | 227 | 77 | 225 | 376 |
| D/D |  | 70 | 21 | 66 | 122 |
| *NFKBIA* (rs696) |  |  |  |  |  |
| GG |  | 216 | 76 | 197 | 298 |
| GA |  | 206 | 61 | 202 | 366 |
| AA |  | 53 | 13 | 59 | 101 |
| *NLRP1* (rs2670660) | |  |  |  |  |
| AA |  | 157 | 51 | 130 | 222 |
| AG |  | 231 | 66 | 233 | 390 |
| GG |  | 89 | 34 | 94 | 154 |
| *NLRP1* (rs878329) |  |  |  |  |  |
| GG |  | 162 | 49 | 128 | 217 |
| GC |  | 231 | 72 | 237 | 394 |
| CC |  | 84 | 29 | 89 | 155 |
| *NLRP3* (rs10754558) | |  |  |  |  |
| CC |  | 178 | 54 | 171 | 294 |
| CG |  | 215 | 74 | 211 | 355 |
| GG |  | 83 | 22 | 70 | 111 |
| *NLRP3* (rs4612666) | |  |  |  |  |
| CC |  | 285 | 89 | 250 | 435 |
| CT |  | 163 | 51 | 165 | 280 |
| TT |  | 29 | 11 | 42 | 53 |
| *PPARG* (rs1801282) | |  |  |  |  |
| CC |  | 355 | 116 | 357 | 548 |
| CG |  | 115 | 33 | 97 | 207 |
| GG |  | 9 | 2 | 4 | 14 |
| *PTPN22* (rs2476601) | |  |  |  |  |
| GG |  | 386 | 123 | 358 | 588 |
| GA |  | 91 | 28 | 92 | 166 |
| AA |  | 2 | 0 | 7 | 11 |
| *SUMO4* (rs237025) |  |  |  |  |  |
| TT |  | 136 | 40 | 117 | 215 |
| TC |  | 232 | 75 | 230 | 362 |
| CC |  | 111 | 36 | 112 | 195 |
| *TBX21* (rs17250932) | |  |  |  |  |
| TT |  | 311 | 101 | 309 | 526 |
| TC |  | 149 | 45 | 131 | 210 |
| CC |  | 17 | 5 | 16 | 32 |
| *TGF-B1* (rs1800469) | |  |  |  |  |
| CC |  | 240 | 73 | 246 | 383 |
| CT |  | 196 | 62 | 184 | 297 |
| TT |  | 40 | 14 | 28 | 86 |
| *TIRAP* (rs8177374) |  |  |  |  |  |
| CC |  | 340 | 108 | 333 | 556 |
| CT |  | 127 | 40 | 120 | 185 |
| TT |  | 10 | 2 | 6 | 21 |
| *TLR1* (rs4833095) |  |  |  |  |  |
| TT |  | 259 | 83 | 276 | 485 |
| TC |  | 188 | 60 | 166 | 261 |
| CC |  | 30 | 8 | 16 | 20 |
| *TLR2* (rs11938228) |  |  |  |  |  |
| CC |  | 217 | 74 | 189 | 327 |
| CA |  | 220 | 65 | 204 | 368 |
| AA |  | 42 | 12 | 66 | 76 |
| *TLR2* (rs1816702) |  |  |  |  |  |
| CC |  | 356 | 120 | 350 | 599 |
| CT |  | 112 | 29 | 94 | 148 |
| TT |  | 11 | 2 | 11 | 10 |
| *TLR2* (rs3804099) |  |  |  |  |  |
| TT |  | 134 | 48 | 142 | 241 |
| TC |  | 238 | 71 | 214 | 393 |
| CC |  | 103 | 32 | 100 | 144 |
| *TLR2* (rs4696480) |  |  |  |  |  |
| AA |  | 123 | 49 | 123 | 199 |
| AT |  | 252 | 75 | 222 | 417 |
| TT |  | 102 | 25 | 113 | 155 |
| *TLR4* (rs12377632) |  |  |  |  |  |
| TT |  | 187 | 55 | 180 | 306 |
| TC |  | 210 | 69 | 189 | 358 |
| CC |  | 76 | 25 | 80 | 102 |
| *TLR4* (rs1554973) |  |  |  |  |  |
| TT |  | 292 | 105 | 280 | 440 |
| TC |  | 161 | 41 | 151 | 272 |
| CC |  | 26 | 5 | 26 | 62 |
| *TLR4* (rs5030728) |  |  |  |  |  |
| GG |  | 221 | 62 | 205 | 359 |
| GA |  | 210 | 66 | 196 | 323 |
| AA |  | 46 | 22 | 55 | 78 |
| *TLR5* (rs5744168) |  |  |  |  |  |
| CC |  | 411 | 127 | 395 | 672 |
| CT |  | 59 | 20 | 62 | 94 |
| TT |  | 5 | 3 | 1 | 5 |
| *TLR5* (rs5744174) |  |  |  |  |  |
| TT |  | 164 | 57 | 138 | 215 |
| TC |  | 233 | 71 | 223 | 399 |
| CC |  | 80 | 22 | 95 | 144 |
| *TLR9* (rs187084) |  |  |  |  |  |
| TT |  | 168 | 50 | 170 | 262 |
| TC |  | 235 | 75 | 201 | 366 |
| CC |  | 73 | 26 | 87 | 142 |
| *TLR9* (rs352139) |  |  |  |  |  |
| GG |  | 135 | 47 | 136 | 255 |
| GA |  | 239 | 72 | 224 | 347 |
| AA |  | 101 | 32 | 95 | 167 |
| *TNF* (rs1800629) |  |  |  |  |  |
| GG |  | 341 | 109 | 320 | 527 |
| GA |  | 126 | 39 | 128 | 223 |
| AA |  | 10 | 2 | 9 | 25 |
| *TNF* (rs361525) |  |  |  |  |  |
| GG |  | 363 | 112 | 381 | 708 |
| GA |  | 108 | 35 | 71 | 60 |
| AA |  | 7 | 3 | 5 | 3 |
| *TNFAIP3* (rs6927172) | |  |  |  |  |
| CC |  | 312 | 97 | 292 | 473 |
| CG |  | 147 | 46 | 140 | 264 |
| GG |  | 18 | 7 | 26 | 40 |
| *TNFRSF1A* (rs4149570) | |  |  |  |  |
| GG |  | 181 | 64 | 164 | 307 |
| GT |  | 222 | 61 | 231 | 355 |
| TT |  | 72 | 24 | 62 | 109 |
| **Abbreviations:** PsO, psoriasis; PsC, cutaneous psoriasis; PsA, psoriatic arthritis; PsC10, patients with PsC followed for ≥10 years. | | | | | |
