# Supplementary material for: Genetic polymorphisms associated with psoriasis and development of psoriatic arthritis in patients with psoriasis
Source: PLoS One. 2018 Feb 1;13(2):e0192010. doi: 10.1371/journal.pone.0192010 (PMC5794107; doi:10.1371/journal.pone.0192010)
Supplement: S3 Table — (DOCX) [file pone.0192010.s003.docx]

| **Supplementary Table 3:** Unadjusted odds ratios (OR) for genotypes studied among healthy controls and patients with psoriasis (PsO), patients with isolated cutaneous psoriasis followed for ≥10 years (PsC10), patients with psoriatic arthritis (PsA), and comparison of PsA with PsC10. | | | | | | | | | | | | | | | | |
| --- | --- | --- | --- | --- | --- | --- | --- | --- | --- | --- | --- | --- | --- | --- | --- | --- |
|  | | | | | **PsO** | | | **PsC10** | | | **PsA** | | | **PsA vs. PsC10** | | |
| Gene (rs-number) | **N**  **^PsO^** | **N**  **^PsC10^** | **N**  **^PsA^** | **N**  **^control^** | OR (95% CI) | *P-*value | q-value | OR (95% CI) | *P-*value | q-value | OR (95% CI) | *P-*value | q-value | OR (95% CI)^a^ | *P-*value | q-value |
| N total | 480 | 151 | 459 | 795 |  |  |  |  |  |  |  |  |  |  |  |  |
| *CARD8* (rs2043211) |  |  |  |  |  |  |  |  |  |  |  |  |  |  |  |  |
| AA | 228 | 69 | 201 | 321 |  |  |  |  |  |  |  |  |  |  |  |  |
| AT or TT vs AA | 250 | 82 | 256 | 436 | 0.81 (0.64–1.02) | 0.07 | 0.59 | 0.87 (0.62–1.24) | 0.46 | 0.86 | 0.94 (0.74–1.19) | 0.59 | 0.92 | 1.07 (0.74–1.55) | 0.71 | 0.95 |
| *CD14* (rs2569190) |  |  |  |  |  |  |  |  |  |  |  |  |  |  |  |  |
| GG | 125 | 36 | 130 | 236 |  |  |  |  |  |  |  |  |  |  |  |  |
| GA or AA vs GG | 348 | 114 | 327 | 530 | 1.24 (0.96–1.60) | 0.09 | 0.59 | 1.41 (0.94–2.11) | 0.09 | 0.59 | 1.12 (0.87–1.44) | 0.38 | 0.82 | 0.79 (0.52–1.22) | 0.29 | 0.79 |
| *IFNG* (rs2430561) |  |  |  |  |  |  |  |  |  |  |  |  |  |  |  |  |
| TT | 131 | 47 | 138 | 199 |  |  |  |  |  |  |  |  |  |  |  |  |
| TA or AA vs TT | 339 | 101 | 318 | 559 | 0.92 (0.71–1.19) | 0.53 | 0.89 | 0.77 (0.52–1.12) | 0.17 | 0.71 | 0.82 (0.63–1.06) | 0.13 | 0.66 | 1.07 (0.72–1.59) | 0.73 | 0.96 |
| *IFNGR1* (rs2234711) |  |  |  |  |  |  |  |  |  |  |  |  |  |  |  |  |
| TT | 168 | 66 | 173 | 290 |  |  |  |  |  |  |  |  |  |  |  |  |
| TC or CC vs TT | 304 | 85 | 281 | 480 | 1.09 (0.86–1.39) | 0.46 | 0.86 | 0.78 (0.55–1.11) | 0.16 | 0.71 | 0.98 (0.77–1.25) | 0.88 | 0.97 | 1.26 (0.87–1.83) | 0.22 | 0.77 |
| *IFNGR2* (rs17882748) |  |  |  |  |  |  |  |  |  |  |  |  |  |  |  |  |
| CC | 135 | 45 | 119 | 199 |  |  |  |  |  |  |  |  |  |  |  |  |
| CT or TT vs CC | 336 | 100 | 339 | 544 | 0.91 (0.70–1.18) | 0.48 | 0.86 | 0.81 (0.55–1.19) | 0.29 | 0.79 | 1.04 (0.79–1.36) | 0.76 | 0.96 | 1.28 (0.85–1.93) | 0.23 | 0.77 |
| *IFNGR2* (rs8126756) |  |  |  |  |  |  |  |  |  |  |  |  |  |  |  |  |
| TT | 349 | 100 | 343 | 553 |  |  |  |  |  |  |  |  |  |  |  |  |
| TC or CC vs TT | 125 | 49 | 113 | 186 | 1.06 (0.82–1.39) | 0.64 | 0.94 | 1.46 (0.99–2.13) | 0.05 | 0.53 | 0.98 (0.75–1.28) | 0.88 | 0.97 | 0.67 (0.45–1.01) | 0.05 | 0.53 |
| *IL1B* (rs1143623) |  |  |  |  |  |  |  |  |  |  |  |  |  |  |  |  |
| GG | 257 | 77 | 229 | 401 |  |  |  |  |  |  |  |  |  |  |  |  |
| GC or CC vs GG | 218 | 72 | 230 | 371 | 0.92 (0.73–1.15) | 0.46 | 0.86 | 1.01 (0.71–1.44) | 0.95 | 0.98 | 1.09 (0.86–1.37) | 0.49 | 0.86 | 1.07 (0.74–1.55) | 0.70 | 0.95 |
| *IL1B* (rs1143627) |  |  |  |  |  |  |  |  |  |  |  |  |  |  |  |  |
| TT | 214 | 67 | 183 | 340 |  |  |  |  |  |  |  |  |  |  |  |  |
| TC or CC vs TT | 264 | 83 | 275 | 436 | 0.96 (0.76–1.21) | 0.74 | 0.96 | 0.97 (0.68–1.37) | 0.85 | 0.97 | 1.17 (0.93–1.48) | 0.19 | 0.74 | 1.21 (0.84–1.76) | 0.31 | 0.82 |
| *IL1B* (rs4848306) |  |  |  |  |  |  |  |  |  |  |  |  |  |  |  |  |
| GG | 141 | 38 | 149 | 246 |  |  |  |  |  |  |  |  |  |  |  |  |
| GA or AA vs GG | 336 | 113 | 308 | 524 | 1.12 (0.87–1.43) | 0.38 | 0.82 | 1.39 (0.94–2.08) | 0.09 | 0.59 | 0.97 (0.76–1.24) | 0.81 | 0.97 | 0.69 (0.46–1.05) | 0.09 | 0.59 |
| *IL1RN* (rs4251961) |  |  |  |  |  |  |  |  |  |  |  |  |  |  |  |  |
| TT | 200 | 64 | 191 | 298 |  |  |  |  |  |  |  |  |  |  |  |  |
| TC or CC vs TT | 277 | 6 | 267 | 472 | 0.87 (0.69–1.10) | 0.26 | 0.78 | 0.85 (0.59–1.21) | 0.36 | 0.82 | 0.88 (0.69–1.12) | 0.29 | 0.79 | 1.04 (0.72–1.51) | 0.84 | 0.97 |
| *IL4R* (rs1805010) |  |  |  |  |  |  |  |  |  |  |  |  |  |  |  |  |
| AA | 127 | 45 | 139 | 209 |  |  |  |  |  |  |  |  |  |  |  |  |
| AG or GG vs AA | 350 | 105 | 318 | 567 | 1.02 (0.79–1.31) | 0.90 | 0.97 | 0.86 (0.59–1.26) | 0.44 | 0.86 | 0.84 (0.65–1.09) | 0.19 | 0.74 | 0.98 (0.66–1.47) | 0.92 | 0.97 |
| *IL6* (rs10499563) |  |  |  |  |  |  |  |  |  |  |  |  |  |  |  |  |
| TT | 309 | 98 | 286 | 476 |  |  |  |  |  |  |  |  |  |  |  |  |
| TC or CC vs TT | 166 | 51 | 170 | 294 | 0.87 (0.69–1.10) | 0.25 | 0.78 | 0.84 (0.58–1.22) | 0.36 | 0.82 | 0.96 (0.76–1.22) | 0.75 | 0.96 | 1.14 (0.77–1.68) | 0.50 | 0.88 |
| *IL6R* (rs4537545) |  |  |  |  |  |  |  |  |  |  |  |  |  |  |  |  |
| CC | 167 | 58 | 168 | 289 |  |  |  |  |  |  |  |  |  |  |  |  |
| CT or TT vs CC | 309 | 90 | 287 | 486 | 1.10 (0.87–1.39) | 0.43 | 0.86 | 0.92 (0.64–1.32) | 0.66 | 0.94 | 1.02 (0.79–1.29) | 0.89 | 0.97 | 1.10 (0.75–1.61) | 0.62 | 0.93 |
| *IL10* (rs1800872) |  |  |  |  |  |  |  |  |  |  |  |  |  |  |  |  |
| CC | 296 | 87 | 278 | 482 |  |  |  |  |  |  |  |  |  |  |  |  |
| CA or AA vs CC | 181 | 64 | 178 | 293 | 1.01 (0.79–1.27) | 0.96 | 0.98 | 1.21 (0.85–1.72) | 0.29 | 0.79 | 1.05 (0.83–1.34) | 0.67 | 0.94 | 0.87 (0.59–1.26) | 0.47 | 0.86 |
| *IL10* (rs3024505) |  |  |  |  |  |  |  |  |  |  |  |  |  |  |  |  |
| CC | 321 | 99 | 309 | 518 |  |  |  |  |  |  |  |  |  |  |  |  |
| CT or TT vs CC | 154 | 52 | 147 | 243 | 1.02 (0.80–1.31) | 0.86 | 0.97 | 1.12 (0.77–1.62) | 0.55 | 0.89 | 1.01 (0.79–1.30) | 0.91 | 0.97 | 0.91 (0.61–1.34) | 0.62 | 0.93 |
| *IL12B* (rs3212217) |  |  |  |  |  |  |  |  |  |  |  |  |  |  |  |  |
| GG | 364 | 110 | 320 | 499 |  |  |  |  |  |  |  |  |  |  |  |  |
| GC or CC vs GG | 115 | 41 | 135 | 260 | 0.61 (0.47–0.89) | **0.0060** | 0.16 | 0.72 (0.48–1.06) | 0.09 | 0.59 | 0.81 (0.63–1.04) | 0.09 | 0.59 | 1.13 (0.75–1.71) | 0.55 | 0.89 |
| *IL12B* (rs6887695) |  |  |  |  |  |  |  |  |  |  |  |  |  |  |  |  |
| GG | 290 | 87 | 243 | 385 |  |  |  |  |  |  |  |  |  |  |  |  |
| GC or CC vs GG | 186 | 63 | 212 | 365 | 0.68 (0.54–0.85) | **0.0010** | 0.047 | 0.76 (0.54–1.09) | 0.14 | 0.67 | 0.92 (0.73–1.16) | 0.48 | 0.86 | 1.20 (0.83–1.75) | 0.33 | 0.82 |
| *IL12RB1* (rs401502) |  |  |  |  |  |  |  |  |  |  |  |  |  |  |  |  |
| CC | 227 | 75 | 206 | 360 |  |  |  |  |  |  |  |  |  |  |  |  |
| CG or GG vs CC | 251 | 75 | 250 | 390 | 1.02 (0.81–1.28) | 0.86 | 0.97 | 0.92 (0.65–1.31) | 0.65 | 0.94 | 1.12 (0.89–1.41) | 0.34 | 0.82 | 1.21 (0.84–1.76) | 0.30 | 0.81 |
| *IL17A* (rs2276913) |  |  |  |  |  |  |  |  |  |  |  |  |  |  |  |  |
| GG | 197 | 62 | 177 | 340 |  |  |  |  |  |  |  |  |  |  |  |  |
| GA or AA vs GG | 281 | 89 | 277 | 431 | 1.13 (0.89–1.42) | 0.32 | 0.82 | 1.13 (0.79–1.61) | 0.49 | 0.86 | 1.23 (0.97–1.56) | 0.08 | 0.59 | 1.09 (0.75–1.59) | 0.65 | 0.94 |
| *IL18* (rs187238) |  |  |  |  |  |  |  |  |  |  |  |  |  |  |  |  |
| GG | 246 | 76 | 241 | 387 |  |  |  |  |  |  |  |  |  |  |  |  |
| GC or CC vs GG | 231 | 74 | 216 | 376 | 0.97 (0.77–1.22) | 0.77 | 0.96 | 1.00 (0.71–1.42) | 0.99 | 0.99 | 0.92 (0.73–1.16) | 0.49 | 0.86 | 0.92 (0.64–1.33) | 0.66 | 0.94 |
| *IL18* (rs1946518) |  |  |  |  |  |  |  |  |  |  |  |  |  |  |  |  |
| CC | 185 | 59 | 166 | 282 |  |  |  |  |  |  |  |  |  |  |  |  |
| CA or AA vs CC | 293 | 92 | 291 | 476 | 0.94 (0.74–1.19) | 0.59 | 0.92 | 0.92 (0.65–1.32) | 0.66 | 0.94 | 1.04 (0.82–1.32) | 0.76 | 0.96 | 1.12 (0.77–1.64) | 0.54 | 0.89 |
| *IL23R* (rs11209026) |  |  |  |  |  |  |  |  |  |  |  |  |  |  |  |  |
| GG | 448 | 144 | 419 | 680 |  |  |  |  |  |  |  |  |  |  |  |  |
| GA or AA vs GG | 31 | 7 | 40 | 94 | 0.50 (0.33–0.76) | **0.0013** | 0.05 | 0.35 (0.16–0.77) | **0.0094** | 0.21 | 0.69 (0.47–1.02) | 0.06 | 0.59 | 1.96 (0.86–4.48) | 0.11 | 0.64 |
| *JAK2* (rs12343867) |  |  |  |  |  |  |  |  |  |  |  |  |  |  |  |  |
| TT | 251 | 75 | 236 | 398 |  |  |  |  |  |  |  |  |  |  |  |  |
| TC or CC vs TT | 224 | 73 | 221 | 360 | 0.99 (0.78–1.24) | 0.91 | 0.97 | 1.08 (0.76–1.53) | 0.68 | 0.94 | 1.04 (0.82–1.31) | 0.77 | 0.96 | 0.96 (0.66–1.39) | 0.84 | 0.97 |
| *LY96* (rs11465996) |  |  |  |  |  |  |  |  |  |  |  |  |  |  |  |  |
| CC | 229 | 62 | 207 | 344 |  |  |  |  |  |  |  |  |  |  |  |  |
| CG or GG vs CC | 250 | 89 | 250 | 418 | 0.89 (0.71–1.29) | 0.36 | 0.82 | 1.18 (0.83–1.68) | 0.36 | 0.82 | 0.99 (0.79–1.25) | 0.96 | 0.98 | 0.84 (0.58–1.22) | 0.36 | 0.82 |
| *MAP3k14* (rs7222094) |  |  |  |  |  |  |  |  |  |  |  |  |  |  |  |  |
| TT | 159 | 51 | 141 | 235 |  |  |  |  |  |  |  |  |  |  |  |  |
| TC or CC vs TT | 318 | 100 | 315 | 530 | 0.89 (0.69–1.13) | 0.34 | 0.82 | 0.87 (0.60–1.26) | 0.46 | 0.86 | 0.99 (0.77–1.27) | 0.94 | 0.98 | 1.14 (0.77–1.69) | 0.51 | 0.88 |
| *NFKB1* (rs28362491) |  |  |  |  |  |  |  |  |  |  |  |  |  |  |  |  |
| I/I | 181 | 53 | 166 | 269 |  |  |  |  |  |  |  |  |  |  |  |  |
| I/D or D/D vs I/I | 297 | 98 | 291 | 498 | 0.89 (0.69–1.12) | 0.32 | 0.82 | 0.99 (0.69–1.44) | 0.99 | 0.99 | 0.95 (0.74–1.21) | 0.66 | 0.94 | 0.95 (0.65–1.39) | 0.79 | 0.97 |
| *NFKBIA* (rs696) |  |  |  |  |  |  |  |  |  |  |  |  |  |  |  |  |
| GG | 216 | 76 | 197 | 298 |  |  |  |  |  |  |  |  |  |  |  |  |
| GA or AA vs GG | 259 | 74 | 261 | 467 | 0.77 (0.61–0.96) | **0.024** | 0.38 | 0.62 (0.44–0.88) | **0.0079** | 0.19 | 0.85 (0.67–1.07) | 0.16 | 0.71 | 1.36 (0.94–1.97) | 0.10 | 0.63 |
| *NLRP1* (rs2670660) |  |  |  |  |  |  |  |  |  |  |  |  |  |  |  |  |
| AA | 157 | 51 | 130 | 222 |  |  |  |  |  |  |  |  |  |  |  |  |
| AG or GG vs AA | 320 | 100 | 327 | 544 | 0.82 (0.65–1.08) | 0.14 | 0.67 | 0.80 (0.55–1.16) | 0.24 | 0.78 | 1.03 (0.79–1.33) | 0.84 | 0.97 | 1.28 (0.87–1.90) | 0.22 | 0.77 |
| *NLRP1* (rs878329) |  |  |  |  |  |  |  |  |  |  |  |  |  |  |  |  |
| GG | 162 | 49 | 128 | 217 |  |  |  |  |  |  |  |  |  |  |  |  |
| GC or CC vs GG | 315 | 101 | 326 | 549 | 0.77 (0.60–0.98) | **0.036** | 0.48 | 0.81 (0.56–1.19) | 0.29 | 0.79 | 1.01 (0.78–1.30) | 0.96 | 0.98 | 1.24 (0.83–1.84) | 0.29 | 0.79 |
| *NLRP3* (rs10754558) |  |  |  |  |  |  |  |  |  |  |  |  |  |  |  |  |
| CC | 178 | 54 | 171 | 294 |  |  |  |  |  |  |  |  |  |  |  |  |
| CG or GG vs CC | 298 | 96 | 281 | 466 | 1.06 (0.83–1.34) | 0.65 | 0.94 | 1.12 (0.78–1.61) | 0.54 | 0.89 | 1.04 (0.82–1.32) | 0.77 | 0.96 | 0.92 (0.63–1.36) | 0.69 | 0.94 |
| *NLRP3* (rs4612666) |  |  |  |  |  |  |  |  |  |  |  |  |  |  |  |  |
| CC | 285 | 89 | 250 | 435 |  |  |  |  |  |  |  |  |  |  |  |  |
| CT or TT vs CC | 192 | 62 | 207 | 333 | 0.88 (0.69–1.11) | 0.28 | 0.79 | 0.91 (0.64–1.29) | 0.60 | 0.93 | 1.08 (0.86–1.37) | 0.51 | 0.88 | 1.19 (0.82–1.73) | 0.36 | 0.82 |
| *PPARG* (rs1801282) |  |  |  |  |  |  |  |  |  |  |  |  |  |  |  |  |
| CC | 355 | 116 | 357 | 548 |  |  |  |  |  |  |  |  |  |  |  |  |
| CG or GG vs CC | 124 | 35 | 101 | 221 | 0.87 (0.67–1.12) | 0.27 | 0.79 | 0.75 (0.49–1.13) | 0.16 | 0.71 | 0.70 (0.54–0.92) | **0.010** | 0.21 | 0.94 (0.61–1.45) | 0.77 | 0.96 |
| *PTPN22* (rs2476601) |  |  |  |  |  |  |  |  |  |  |  |  |  |  |  |  |
| GG | 386 | 123 | 358 | 588 |  |  |  |  |  |  |  |  |  |  |  |  |
| GA or AA vs GG | 93 | 28 | 99 | 177 | 0.80 (0.60–1.06) | 0.12 | 0.65 | 0.76 (0.49–1.18) | 0.22 | 0.77 | 0.92 (0.69–1.21) | 0.55 | 0.89 | 1.21 (0.76–1.94) | 0.41 | 0.86 |
| *SUMO4* (rs237025) |  |  |  |  |  |  |  |  |  |  |  |  |  |  |  |  |
| TT | 136 | 40 | 117 | 215 |  |  |  |  |  |  |  |  |  |  |  |  |
| TC or CC vs TT | 343 | 111 | 342 | 557 | 0.97 (0.76–1.25) | 0.84 | 0.97 | 1.07 (0.72–1.59) | 0.73 | 0.96 | 1.13 (0.87–1.47) | 0.37 | 0.82 | 1.05 (0.69–1.59) | 0.81 | 0.97 |
| *TBX21* (rs17250932) |  |  |  |  |  |  |  |  |  |  |  |  |  |  |  |  |
| TT | 311 | 101 | 309 | 526 |  |  |  |  |  |  |  |  |  |  |  |  |
| TC or CC vs TT | 166 | 50 | 147 | 242 | 1.16 (0.91–1.48) | 0.23 | 0.77 | 1.08 (0.74–1.56) | 0.69 | 0.94 | 1.03 (0.81–1.33) | 0.79 | 0.97 | 0.96 (0.65–1.42) | 0.84 | 0.97 |
| *TGF–B1* (rs1800469) |  |  |  |  |  |  |  |  |  |  |  |  |  |  |  |  |
| CC | 240 | 73 | 246 | 383 |  |  |  |  |  |  |  |  |  |  |  |  |
| CT or TT vs CC | 236 | 76 | 212 | 383 | 0.98 (0.78–1.24) | 0.89 | 0.97 | 1.04 (0.73–1.48) | 0.82 | 0.97 | 0.86 (0.68–1.09) | 0.21 | 0.77 | 0.83 (0.57–1.19) | 0.32 | 0.82 |
| *TIRAP* (rs8177374) |  |  |  |  |  |  |  |  |  |  |  |  |  |  |  |  |
| CC | 340 | 108 | 333 | 556 |  |  |  |  |  |  |  |  |  |  |  |  |
| CT or TT vs CC | 137 | 42 | 126 | 206 | 1.09 (0.84–1.40) | 0.52 | 0.89 | 1.05 (0.71–1.55) | 0.81 | 0.97 | 1.02 (0.79–1.32) | 0.87 | 0.97 | 0.97 (0.64–1.47) | 0.89 | 0.97 |
| *TLR1* (rs4833095) |  |  |  |  |  |  |  |  |  |  |  |  |  |  |  |  |
| TT | 259 | 83 | 276 | 485 |  |  |  |  |  |  |  |  |  |  |  |  |
| TC or CC vs TT | 218 | 68 | 182 | 281 | 1.45 (1.15–1.83) | **0.0016** | 0.06 | 1.41 (0.99–2.01) | 0.05 | 0.53 | 1.14 (0.89–1.44) | 0.29 | 0.79 | 0.80 (0.56–1.17) | 0.25 | 0.78 |
| *TLR2* (rs11938228) |  |  |  |  |  |  |  |  |  |  |  |  |  |  |  |  |
| CC | 217 | 74 | 189 | 327 |  |  |  |  |  |  |  |  |  |  |  |  |
| CA or AA vs CC | 262 | 77 | 270 | 444 | 0.89 (0.71–1.12) | 0.32 | 0.82 | 0.77 (0.54–1.09) | 0.14 | 0.67 | 1.05 (0.83–1.33) | 0.67 | 0.94 | 1.37 (0.95–1.99) | 0.09 | 0.59 |
| *TLR2* (rs1816702) |  |  |  |  |  |  |  |  |  |  |  |  |  |  |  |  |
| CC | 356 | 120 | 350 | 599 |  |  |  |  |  |  |  |  |  |  |  |  |
| CT or TT vs CC | 123 | 31 | 105 | 158 | 1.31 (1.00–1.72) | **0.049** | 0.53 | 0.98 (0.64–1.51) | 0.92 | 0.97 | 1.14 (0.86–1.50) | 0.37 | 0.82 | 1.16 (0.74–1.82) | 0.52 | 0.89 |
| *TLR2* (rs3804099) |  |  |  |  |  |  |  |  |  |  |  |  |  |  |  |  |
| TT | 134 | 48 | 142 | 241 |  |  |  |  |  |  |  |  |  |  |  |  |
| TC or CC vs TT | 341 | 103 | 314 | 537 | 1.14 (0.89–1.47) | 0.29 | 0.79 | 0.96 (0.66–1.40) | 0.84 | 0.97 | 0.99 (0.77–1.27) | 0.95 | 0.98 | 1.03 (0.69–1.53) | 0.88 | 0.97 |
| *TLR2* (rs4696480) |  |  |  |  |  |  |  |  |  |  |  |  |  |  |  |  |
| AA | 123 | 49 | 123 | 199 |  |  |  |  |  |  |  |  |  |  |  |  |
| AT or TT vs AA | 354 | 100 | 335 | 572 | 1.00 (0.77–1.29) | 0.99 | 0.99 | 0.71 (0.49–1.04) | 0.08 | 0.59 | 0.95 (0.73–1.23) | 0.69 | 0.94 | 1.33 (0.89–1.99) | 0.16 | 0.71 |
| *TLR4* (rs12377632) |  |  |  |  |  |  |  |  |  |  |  |  |  |  |  |  |
| TT | 187 | 55 | 180 | 306 |  |  |  |  |  |  |  |  |  |  |  |  |
| TC or CC vs TT | 286 | 94 | 269 | 460 | 1.02 (0.80–1.29) | 0.89 | 0.97 | 1.14 (0.79–1.63) | 0.49 | 0.86 | 0.99 (0.78–1.26) | 0.96 | 0.98 | 0.87 (0.59–1.28) | 0.49 | 0.86 |
| *TLR4* (rs1554973) |  |  |  |  |  |  |  |  |  |  |  |  |  |  |  |  |
| TT | 292 | 105 | 280 | 440 |  |  |  |  |  |  |  |  |  |  |  |  |
| TC or CC vs TT | 187 | 46 | 177 | 334 | 0.84 (0.67–1.06) | 0.15 | 0.69 | 0.58 (0.39–0.84) | **0.0040** | 0.12 | 0.83 (0.66–1.05) | 0.13 | 0.66 | 1.44 (0.97–2.14) | 0.07 | 0.59 |
| *TLR4* (rs5030728) |  |  |  |  |  |  |  |  |  |  |  |  |  |  |  |  |
| GG | 221 | 62 | 205 | 359 |  |  |  |  |  |  |  |  |  |  |  |  |
| GA or AA vs GG | 256 | 88 | 251 | 401 | 1.04 (0.82–1.30) | 0.76 | 0.96 | 1.27 (0.89–1.81) | 0.19 | 0.74 | 1.09 (0.87–1.38) | 0.44 | 0.86 | 0.86 (0.59–1.25) | 0.44 | 0.86 |
| *TLR5* (rs5744168) |  |  |  |  |  |  |  |  |  |  |  |  |  |  |  |  |
| CC | 411 | 127 | 395 | 672 |  |  |  |  |  |  |  |  |  |  |  |  |
| CT or TT vs CC | 64 | 23 | 63 | 99 | 1.06 (0.75–1.48) | 0.75 | 0.96 | 1.23 (0.75–2.01) | 0.41 | 0.86 | 1.08 (0.77–1.52) | 0.65 | 0.94 | 0.88 (0.52–1.48) | 0.63 | 0.93 |
| *TLR5* (rs5744174) |  |  |  |  |  |  |  |  |  |  |  |  |  |  |  |  |
| TT | 164 | 57 | 138 | 215 |  |  |  |  |  |  |  |  |  |  |  |  |
| TC or CC vs TT | 313 | 93 | 318 | 543 | 0.76 (0.59–0.97) | **0.026** | 0.39 | 0.65 (0.45–0.93) | **0.019** | 0.32 | 0.91 (0.71–1.18) | 0.48 | 0.86 | 1.41 (0.96–2.08) | 0.08 | 0.59 |
| *TLR9* (rs187084) |  |  |  |  |  |  |  |  |  |  |  |  |  |  |  |  |
| TT | 168 | 50 | 170 | 262 |  |  |  |  |  |  |  |  |  |  |  |  |
| TC or CC vs TT | 308 | 101 | 288 | 508 | 0.95 (0.74–1.20) | 0.65 | 0.94 | 1.04 (0.72–1.51) | 0.83 | 0.97 | 0.87 (0.69–1.11) | 0.27 | 0.79 | 0.84 (0.57–1.24) | 0.37 | 0.82 |
| *TLR9* (rs352139) |  |  |  |  |  |  |  |  |  |  |  |  |  |  |  |  |
| GG | 135 | 47 | 136 | 255 |  |  |  |  |  |  |  |  |  |  |  |  |
| GA or AA vs GG | 340 | 104 | 319 | 514 | 1.25 (0.97–1.60) | 0.08 | 0.59 | 1.09 (0.75–1.59) | 0.63 | 0.93 | 1.16 (0.91–1.49) | 0.24 | 0.78 | 1.06 (0.71–1.58) | 0.77 | 0.96 |
| *TNF* (rs1800629) |  |  |  |  |  |  |  |  |  |  |  |  |  |  |  |  |
| GG | 341 | 109 | 320 | 527 |  |  |  |  |  |  |  |  |  |  |  |  |
| GA or AA vs GG | 136 | 41 | 137 | 248 | 0.85 (0.66–1.09) | 0.19 | 0.74 | 0.79 (0.54–1.18) | 0.26 | 0.78 | 0.91 (0.71–1.17) | 0.46 | 0.86 | 1.14 (0.75–1.72) | 0.54 | 0.89 |
| *TNF* (rs361525) |  |  |  |  |  |  |  |  |  |  |  |  |  |  |  |  |
| GG | 363 | 112 | 381 | 708 |  |  |  |  |  |  |  |  |  |  |  |  |
| GA or AA vs GG | 115 | 38 | 76 | 63 | 3.56 (2.55–4.96) | **<10^–6^** | 0.00011 | 3.81 (2.43–5.98) | **<10^–6^** | 0.00011 | 2.24 (1.57–3.20) | **9*10^–6^** | 0.00064 | 0.59 (0.38–0.92) | **0.019** | 0.32 |
| *TNFAIP3* (rs6927172) |  |  |  |  |  |  |  |  |  |  |  |  |  |  |  |  |
| CC | 312 | 97 | 292 | 473 |  |  |  |  |  |  |  |  |  |  |  |  |
| CG or GG vs CC | 165 | 53 | 166 | 304 | 0.82 (0.65–1.04) | 0.11 | 0.64 | 0.85 (0.59–1.22) | 0.38 | 0.82 | 0.88 (0.69–1.12) | 0.31 | 0.82 | 1.04 (0.71–1.53) | 0.84 | 0.97 |
| *TNFRSF1A* (rs4149570) | |  |  |  |  |  |  |  |  |  |  |  |  |  |  |  |
| GG | 181 | 64 | 164 | 307 |  |  |  |  |  |  |  |  |  |  |  |  |
| GA or AA vs GG | 294 | 85 | 293 | 464 | 1.07 (0.85–1.36) | 0.55 | 0.89 | 0.88 (0.62–1.25) | 0.48 | 0.86 | 1.18 (0.93–1.50) | 0.17 | 0.71 | 1.35 (0.92–1.96) | 0.12 | 0.65 |
|  |  |  |  |  |  |  |  |  |  |  |  |  |  |  |  |  |
| **Abbreviations:** CI, confidence interval; OR, odds ratio. PsO, psoriasis, PsC, isolated cutaneous psoriasis; PsA, psoriatic arthritis; PsC10, patients with PsC followed for ≥10 years. OR, odds ratio (> 1.00 associated with increased risk of disease).  ^a^OR >1.00 associated with increased risk of PsA compared to PsC10.  All *P-*values < 0.05 are marked in bold.  All q-values < 0.05 are underlined. | | | | | | | | | | | | | | | | |
